# Treatment of Persistent Large Gastrocutaneous Fistulas After Bariatric Surgery: Preliminary Experience with Endoscopic Kehr’s T-Tube Placement

**DOI:** 10.1007/s11695-022-05935-y

**Published:** 2022-02-09

**Authors:** Arnaud Liagre, Michel Queralto, Jonathan Levy, Jean Marc Combis, Paulo Peireira, Jane N. Buchwald, Gildas Juglard, Niccolò Petrucciani, Francesco Martini

**Affiliations:** 1 Clinique Des Cedres, Bariatric Surgery Unit, Ramsay Générale de Santé, Cornebarrieu, France; 2 Clinique Des Cedres, Gastrointestinal Endoscopy Unit, Cornebarrieu, France; 3 Clinique A. Paré, Gastrointestinal Endoscopy Unit, Toulouse, France; 4 Clinique A. Paré, Bariatric Surgery Unit, Toulouse, France; 5 Division of Scientific Research Writing, Medwrite Medical Communications, Maiden Rock, WI 54750 USA; 6 Department of Medical and Surgical Sciences and Translational Medicine, Faculty of Medicine and Psychology, St Andrea Hospital, Sapienza University, Rome, Italy

**Keywords:** Bariatric surgery, Endoscopy, Leak, Large gastrocutaneous fistula, Kehr's T-tube, Pigtail drain

## Abstract

**Purpose:**

Post-bariatric surgery gastrocutaneous fistula is a chronic leak with an incidence of 1.7 to 4.0% and no standardized management. A large gastrocutaneous fistula (LGCF) is not indicated for treatment with pigtail drains. We aimed to evaluate results of a novel treatment using endoscopic Kehr’s T-tube placement.

**Methods:**

Only patients with a postoperative LGCF duration of > 10 days and a flow rate of > 50 cc by external drainage after revisional surgery for sepsis were included. Endoscopic placement of Kehr’s T-tube was performed. Patients had been reoperated with wash and drainage for severe sepsis after initial bariatric surgery in which no fistula had been discovered. Patients not reoperated, or with a fistula requiring intraoperative Kehr’s T-tube placement, or a pigtail drain were excluded. Primary outcomes were endoscopic characteristics and results (LGCF closure rate, Kehr T-tube retention time, etc.).

**Results:**

The study group included 12 women, 2 men; body mass index 43.1 ± 4.5 kg/m^2^. Interventions were SG (7), RYGB (2), OAGB (4), and SADI-S (1). Endoscopic assessment was carried out after a mean of 33.2 ± 44.3 days after the bariatric procedure. The mean fistula orifice diameter was 2.0 ± 0.9 cm. Kehr’s T-tube was positioned at a mean 51.5 ± 54.8 days after the bariatric procedure. T-tube tolerance was excellent. Mean additional days: hospitalization, 34.4 ± 27.0; T-tube retention, 86.4 ± 73.1; fistula healing, 139.9 ± 111.5, LGCF closure rate, 92.9%. Complications: 1 pulmonary embolism, 2 T-tube migrations,1 drain-path bleed, 1 skin abscess. No mortality.

**Conclusions:**

Endoscopic Kehr’s T-tube placement was successful in closing persistent post-bariatric surgery LGCF in 92.9% of patients.

**Graphical abstract:**

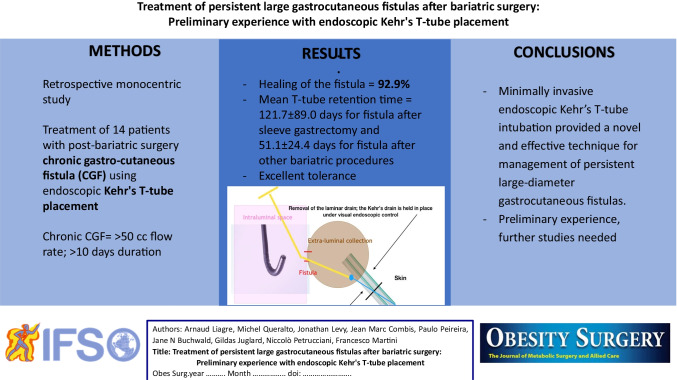

## Introduction

Bariatric procedures affect significant weight loss and comorbidity reduction with a low mortality (0.1%) and overall complications of around 4.0% [1]. Leak leading to abscess or fistula collection is a major adverse event. A conservative nonsurgical endoscopic or radiological approach is usually effective in managing smaller leaks in the short term; however, a chronic large-diameter leak with a fistulous tract is extremely challenging to resolve and may require surgery and extended hospitalization [2].

**Fig. 1:**
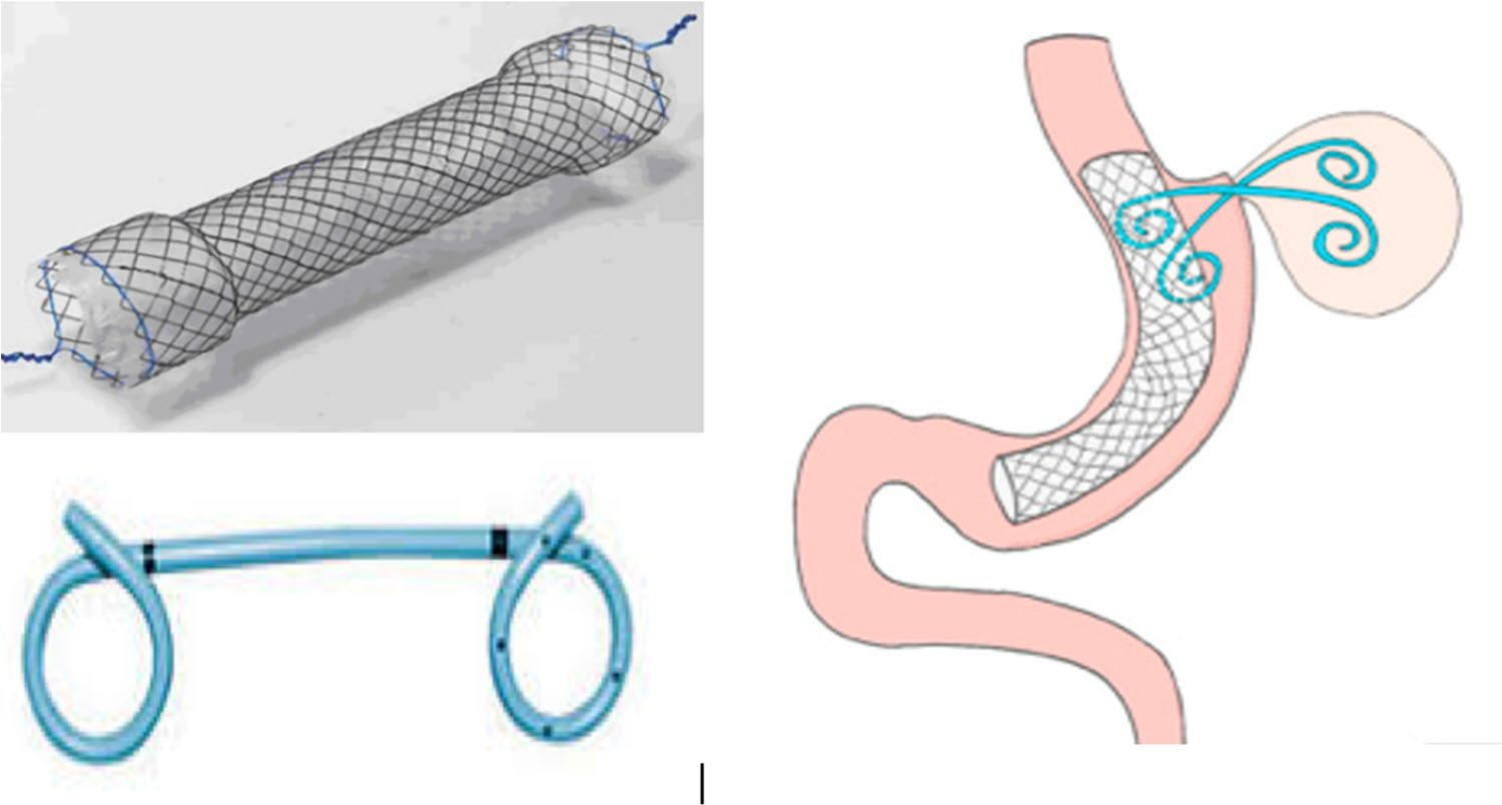
Stent and pigtail drain for closing post-bariatric surgery fistulas

Incidence of leak in patients following Roux-en-Y gastric bypass (RYGB) is approximately 0.6 to 5.6% [3], in post-sleeve gastrectomy (SG), 1.0 to 7.0% [4], and in one-anastomosis gastric bypass (OAGB), 0.1 to 1.5% [5]. A recent American Society for Metabolic and Bariatric Surgery statement suggests that available data do not favor one leak closure treatment over another [6]. The American Gastroenterological Association noted that metallic or plastic stents, pigtail drains, clips, tissue sealants, balloon dilatation, and combination therapies are not always well tolerated and carry a high migration rate [7]. Pigtail drains are often employed as the solution for small leak management but are often ineffective in large-diameter leak resolution (Fig. 1). Primary closure of chronic fistulas with ≥ 2-cm orifices is often unsuccessful, and there is no standardized endoscopic approach [8].

The large gastrocutaneous fistula (LGCF) is a particularly malignant chronic wide-orifice leak that directs gastric spillage through the fistulous tract to an opening in the skin. LGCFs occur in 0.5 to 3.9% of patients who undergo gastrointestinal (GI) surgery, and in 1.7 to 4.0% of fistula cases following bariatric procedures [9, 10]. Advanced expertise is required to resolve LGCFs, which are associated with up to 80.0% mortality in patients undergoing GI surgery [10]. LGCF management following bariatric surgery is not standardized.

**Fig. 2:**
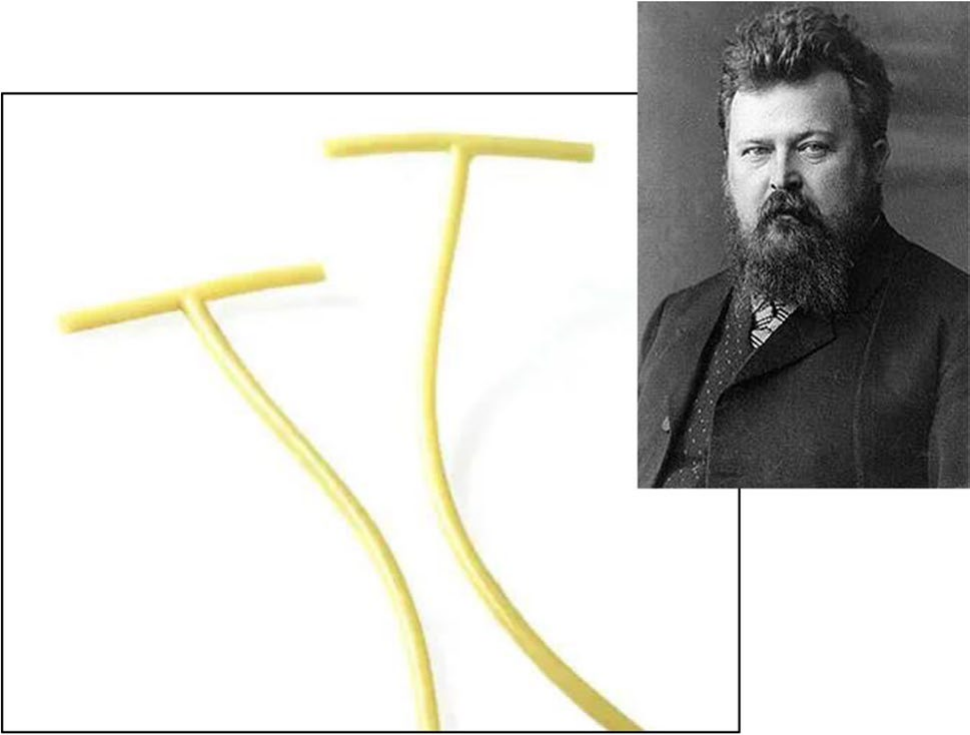
Kehr’s T-tube for gastrocutaneous fistulas. Hans Kehr, professor of surgery and inventor (1862–1916)

The rubber biliary drainage tube developed in 1897 by Hans Kehr [11] (Kehr's T-tube/drain) has been employed for > 100 years in choledochal diseases and as a method adopted for severe esophageal wound treatment [12] (Fig. 2). In addition to the utility of its "T” shape for fastening, the contemporary latex tube material instigates tissue inflammation that expedites more rapid healing than other types of drains in approximately 10 days. Our center previously reported favorable outcomes with laparoscopic intubation of Kehr's T-tube in post-OAGB large-orifice fistulas at the gastrojejunal anastomosis (GJA). This approach obviated RYGB conversion [13].

The study aim was to present preliminary results of LGCF treatment with Kehr's T-tube using endoscopy. We examined outcomes of all persistent LGCFs treated with this novel methodology following OAGB, SG, RYGB, and single-anastomosis duodenoileal bypass with sleeve gastrectomy (SADI-S).

## Materials and Methods

### Study Design and Participants

This study design was a monocentric retrospective analysis of prospectively collected data in a long-term database. We evaluated the data describing outcomes of a novel approach to treat post-bariatric surgery LGCFs using an endoscopically placed Kehr’s T-tube. The study was approved by our Institutional Review Board and was registered as IORG-IRB: IORG0009085 IRB00010835 COS-RGDS-2020–03-004-LIAGRE-A.

### Inclusion and Consent

Patients were included based on having undergone a primary bariatric procedure in our center, and/or being referred to us for advanced postoperative care or a revisional bariatric procedure. Only patients with a postoperative LGCF duration > 10 days and a flow rate > 50 cc by external drainage after revisional surgery for sepsis were included. Kehr’s endoscopic T-tube placement was performed in these cases. These patients had been reoperated for severe sepsis after initial bariatric surgery, and no fistula had been discovered; a simple wash and drainage had been performed. Patients not reoperated, or with a fistula requiring intraoperative Kehr T-tube placement, or with a fistula requiring a pigtail drain were excluded. All participants had completed an informed consent process to be enrolled for a bariatric procedure.

### “Rendezvous” Technique

**Fig. 3:**
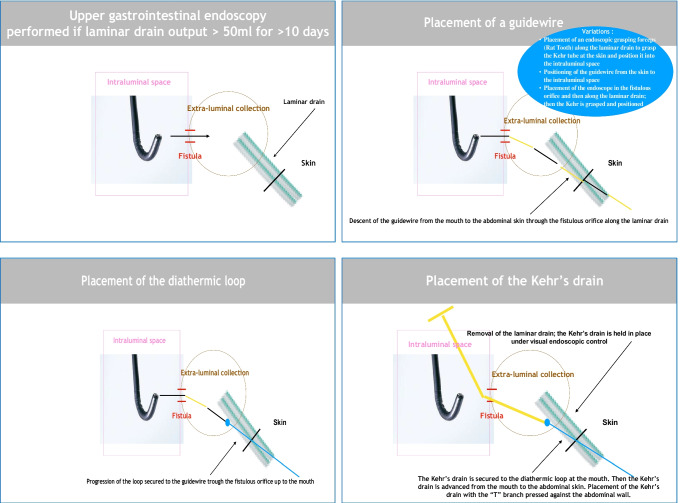
Kehr’s T-tube placement technique for post-bariatric surgery large gastrocutaneous fistulas. **(a)** The guidewire was inserted from the mouth to the stomach and through the fistula drainage pathway to the skin opening. **(b)** A diathermic loop was fixed to the guidewire and progressed retrograde through the fistula from the skin opening up to the mouth

Treatment of the LGCF was performed under general anesthesia using Kehr T-tube placement. The LGCF orifice was localized endoscopically. A Jagwire® guidewire (Boston Scientific, St. Paul, MN) was inserted from the mouth to the stomach and through the fistula drainage pathway to the skin opening along the surgical drain (Fig. 3a). An Exacto® Steris diathermic loop was attached to the guidewire at the skin fistula orifice and progressed up to the mouth in the opposite direction of the guidewire. The loop of the Kehr T-tube® Coloplast 12 or 14 Fr was attached and progressed from the mouth to the skin opening of the fistula to position the “T” in the digestive lumen pressed against the wall. The surgical drain was removed while maintaining the T-tube in place and with the external drainage portion affixed to the skin (Fig. 3b).

Early morbidity following a diagnosed leak treatment was defined as a 90-day complication. A major early morbidity was defined as a grade > IIIa adverse event by Clavien-Dindo classification.

### Technical T-Tube Placement Variations

The standard setup is to use the guidewire and then the diathermic loop which catches the T-tube. The approach is applicable with or without the presence of a drain and in the instance of a small cutaneous orifice. The guidewire can be placed from inside the intestinal lumen to the outside of the skin, or conversely, from the cutaneous opening toward the intestinal lumen. If visualization of the surgical drain is possible during endoscopy, a technical variant that saves endoscopic time consists of advancing an Olympus-type clamp (Fg-53sx-1 Rat Tooth 8-mm Reusable Gripper®) in the intestinal lumen through the fistula along the drain toward the cutaneous opening and directly catching the T-tube, then advancing it in the opposite direction from the skin opening toward the interior of the intestinal lumen.

Another technical variation is possible when the surgical drain is absent due to ablation: If there is a large cutaneous opening to the skin, the guidewire can be installed directly by advancing the fiberscope trans-fistula and attaching the T-tube to the fiberscope. In the case of an abscessed cavity, a nasocystic drain (Liguory®, Cook Medical) was put in place before the T-tube to wash continuously for a few days. Patients were allowed to drink water in order to wash the fistula path, and a second endoscopy was required for placement of the T-tube.

### Follow-up Management (Surgical and Endoscopic)

A gradual progression to oral feeding was initiated. Spontaneously resolving suppuration was usual around the T-tube for a several days. After a few days, the T-tube was clamped. T-tube removal was performed on an outpatient basis.

### Statistical Analysis

Analyses were performed with the SPSS statistical package (version 27.0; IBM, Chicago, IL). Quantitative variables were reported as mean ± SD, range, and median and interquartile range (IQR). Qualitative variables were reported as frequencies (Fisher’s exact test). Between-group comparisons along quantitative variables were performed with the Mann–Whitney *U* test. All tests were two-tailed; statistical significance was *p* < 0.05.

## Results

Fourteen patients were included in the study who suffered from a persistent postoperative gastrocutaneous/duodenocutaneous fistula (> 10 days) with a flow rate through the fistula of > 50 cc. The study was composed of 12 women and 2 men; mean age 44.9 ± 11.3 years (28.0–69.0), body mass index (BMI) 43.1 ± 4.5 kg/m^2^ (34.0–50.0), and weight of 118.9 ± 20.6 kg (84.0–156.0) at the time of the bariatric procedure (Table 1). Interventions performed in our hospital were 7 SG, 2 RYGB, 4 OAGB, and 1 SADI-S. Five patients (36.0%) had previous bariatric procedures (Mason *n* = 2; adjustable gastric band [AGB] with intragastric migration *n* = 1; AGB followed by SG *n* = 1, AGB *n* = 1). In addition, 7 patients had undergone a primary bariatric procedure in another center.

**Table 1:** Patient characteristics

	Total sample (*n* = 14) (12 female, 2 male)		SG (*n* = 7) (6 female, 1 male)		Other procedures (*n* = 7) (6 female, 1 male)		*p* value*
	Mean ± SD (range)	Median (IQR)	Mean ± SD (range)	Median (IQR)	Mean ± SD (range)	Median (IQR)	
Age, years	44.9 ± 11.3 (28.0–69.0)	44.5 (37.0–53.0)	41.7 ± 14.2 (28.0–69.0)	38.0 (32.0–52.0)	48.1 ± 7.1 (40.0–60.0)	46.0 (44.0–56.0)	.128
Height, m	1.66 ± 0.1 (1.52–1.82)	1.67 (1.59–1.70)	1.64 ± 0.1 (1.57–1.70)	1.65 (1.60–1.69)	1.67 ± 0.1 (1.52–1.82)	1.68 (1.56–1.78)	.731
Weight, kg	118.9 ± 20.6 (84.0–156.0)	119.0 (104.3–132.5)	112.9 ± 10.2 (102.0–130.0)	110.0 (105.0–120.0)	124.9 ± 27.1 (84.0–156.0)	125.0 (95.0–150.0)	.318
BMI, kg/m^2^	43.1 ± 4.5 (34.0–50.0)	42.5 (40.8–46.5)	41.6 ± 2.5 (38.0–46.0)	41.0 (40.0–43.0)	44.6 ± 5.7 (34.0–50.0)	45.0 (41.0–50.0)	.165

^*^Mann–Whitney *U* test; comparison between sleeve gastrectomy (SG) vs other procedures

At a mean of 9.2 ± 7.4 days from the first procedure (postoperative day [POD] 2–23), 13/14 patients underwent laparoscopic reoperation for severe sepsis. In these patients, no fistulous orifice had been identified intraoperatively; therefore, washing of the abdominal cavity and drainage were performed. The one patient (of 14 studied) in whom a drain was placed during the bariatric procedure (RYGB) presented with fistula on POD 6.

Endoscopic assessment was carried out in our referral center for persistent high flow of digestive or purulent liquid through the drains after a mean of 33.2 ± 44.3 days (median: 15.5 days), between POD 12 and POD 180 (Table 2). A mean fistula orifice diameter of 2.0 ± 0.9 cm (0.5–3.0) on the gastric tube or on the duodenum was observed. In nine patients, a Liguory nasocystic drain (Cook Medical, Limerick, Ireland) with lavage was placed before (*n* = 6) or at the same time of the placement of the T-tube (*n* = 3) to optimize washing of the extraluminal abscess cavity. Mean washing time was 8.4 ± 4.4 days (3.0–15.0) (Table 2).

**Table 2:** Characteristics of endoscopy

	Total sample (*n* = 14) (12 female, 2 male)		SG (*n* = 7) (6 female, 1 male)		Other procedures (*n* = 7) (6 female, 1 male)		*p* value
	Mean ± SD (range)	Median (IQR)	Mean ± SD (range)	Median (IQR)	Mean ± SD (range)	Median (IQR)	
Days between bariatric proc. and endoscopic assessment	33.2 ± 44.3 (12.0–180.0)	15.5 (14.0–35.0)	45.1 ± 60.5 (14.0–180.0)	16.0 (15.0–44.0)	21.3 ± 15.7 (12.0–56.0)	14.0 (13.0–20.0)	.259*
Days of washing w/external drain	8.4 ± 4.4 (3.0–15.0)	7.0 (5.0–12.5)	8.5 ± 5.0 (3.0–15.0)	8.0 (4.0–13.5)	8.4 ± 4.5 (3.0–15.0)	7.0 (5.0–12.5)	.990*
Days between bariatric proc. and Kehr	51.5 ± 54.8 (12.0–200.0)	27.0 (19.0–66.3)	77.6 ± 68.3 (20.0–200.0)	66.0 (22.0–140.0)	25.4 ± 16.1 (12.0–59.0)	22.0 (14.0–29.0)	.053*
Fistula size, cm	2.0 ± 0.9 (0.5–3.0)	2.0 (1.4–3.0)	1.9 ± 1.2 (0.5–3.0)	2.0 (0.5–3.0)	2.1 ± 0.5 (1.5–3.0)	2.0 (2.0–2.5)	.805*
Fistula location	Tube vertex: *n* = 11 Duodenum: *n* = 1 Anastomosis: *n* = 1 Middle stomach: *n* = 1		Tube vertex: *n* = 6 Duodenum: *n* = 0 Anastomosis: *n* = 0 Middle stomach: *n* = 1		Tube vertex: *n* = 5 Duodenum: *n* = 1 Anastomosis: *n* = 1 Middle stomach: *n* = 0		.990†
Route of gastrocutan-eous fistula	Simple: *n* = 12 Gastrocutaneous and trans-diaphragmatic complex: *n* = 2		Simple: *n* = 5 Gastrocutaneous and trans-diaphragmatic complex: *n* = 2		Simple: *n* = 7 Gastrocutaneous and trans-diaphragmatic complex: *n* = 0		.462†
Internal/external drainage with wash at same or different time of Kehr	Yes, with Kehr at different time: *n* = 6 Yes, with Kehr at same time: *n* = 3 No: *n* = 5		Yes, with Kehr at different time: *n* = 3 Yes, with Kehr at same time: *n* = 1 No: *n* = 3		Yes, with Kehr at different time: *n* = 3 Yes, with Kehr at same time: *n* = 2 No: *n* = 2		.990†

^*^Mann–Whitney *U* test, comparison between sleeve gastrectomy (SG) vs other procedures

^†^Fisher’s exact test, comparison between SG vs other procedures

Twelve patients had simple LGCFs; two, complex LGCFs (gastrocutaneous and transdiaphragmatic with gastrobronchial fistula). Three patients received ineffective endoscopic procedures before their prior drain placement (i.e., pigtail ± Over-the-Scope Clip [OTSC®, Ovesco Endoscopy GmbH, Tübingen, Germany] ± gluing). To treat LGCFs, the T-tube was placed at a mean of 51.5 ± 54.8 days (median: 27.0 days) between POD 12 and 200 in accord with the increase in size of the fistula and flow rate through the drain. There were no significant between-group differences (i.e., SG vs other procedures) in endoscopic characteristics (Table 2).

Transient leakage or purulent discharge around the T-tube was typical for several days and then typically dried up. Patients were fed a liquid diet for several days and then progressed to solid foods. Clinical T-tube tolerance was perfect under a simple dressing. Dietary quality of life was good due to the flexible nature of the prosthesis. Withdrawal of the drain was made after consultation.

Mean additional hospitalization time per bariatric procedure was 34.4 ± 27.0 days (median: 29.0; IQR: 8.0–49.0); mean T-tube retention was 86.4 ± 73.1 days (66.5; 40.0–111.5) (Table 3). As in choledochal surgery, no strictures have occurred after using the Kehr T-tube. The mean time of complete fistula healing (without anastomotic stenosis) in 13/14 cases was 139.9 ± 111.5 days (84.0; 58.0–241.0) calculated from the time of the bariatric procedure. Mean T-tube retention time was 121.7 ± 89.0 days (98.0; 67.0–163.0) with SG (a high-pressure procedure) and 51.1 ± 24.4 days (45.0; 37.0–66.0) for the other (low-pressure) procedures (*p* = 0.053). There were no significant between-group differences in endoscopic T-tube results (Table 3). All patients were followed up between 24 and 72 months after Kehr’s T-tube removal: There was no LGCF recurrence.

**Table 3:** Results of endoscopic Kehr drainage

	Total sample (*n* = 14) (12 female, 2 male)		Sleeve (*n* = 7) (6 female, 1 male)		Other (*n* = 7) (6 female, 1 male)		*p* value
	Mean ± SD (range)	Median (IQR)	Mean ± SD (range)	Median (IQR)	Mean ± SD (range)	Median (IQR)	
Days with Kehr retention	86.4 ± 73.1 (15.0–289.0)	66.5 (40.0–111.5)	121.7 ± 89.9 (15.0–289.0)	98.0 (67.0–163.0)	51.1 ± 24.4 (21.0–97.0)	45.0 (37.0–66.0)	.053*
Days between bariatric proc. and fistula closure	139.9 ± 111.5 (40.0–360.0)	84.0 (58.0–241.0)	214.7 ± 129.4 (40.0–360.0)	241.0 (72.3–332.3)	75.7 ± 22.7 (50.0–115.0)	77.0 (54.0–88.0)	.138*
Addt’l days of hospital stay for fistula mgmt	34.4 ± 27.0 (2.0–90.0)	29.0 (8.0–49.0)	35.1 ± 36.1 (2.0–90.0)	28.0 (5.0–80.0)	33.6 ± 16.5 (8.0–61.0)	30.0 (27.0–45.0)	.710*
Morbidity	Pulmonary embolism: *n* = 1 Bleeding fistula/transfusion path: *n* = 1 Kehr migration: *n* = 2 Abscess at skin, drained in consultation: *n* = 1		Pulmonary embolism: *n* = 0 Bleeding fistula/transfusion path: *n* = 0 Kehr migration: *n* = 1 Abscess at skin, drained in consultation: *n* = 0		Pulmonary embolism: *n* = 1 Bleeding fistula/transfusion path: *n* = 1 Kehr migration: *n* = 1 Abscess at skin, drained in consultation: *n* = 1		.990†
Addt’l endo-scopic proced. after Kehr	Pigtail drain: *n* = 2		Pigtail drain: *n* = 2		Pigtail drain: *n* = 0		.462†
Fistula closure	Yes: *n* = 13		Yes: *n* = 6		Yes: *n* = 7		.990†

^*^Mann–Whitney *U* test, comparison between sleeve gastrectomy (SG) vs other procedures

^†^Fisher’s exact test, comparison between SG vs other procedures

### Complications

Five complications were observed: 1 pulmonary embolism; 1 bleeding in the path of the drain requiring a blood transfusion; 2 T-tube migrations (in one case aspirated inward and discharged anally; in the other, falling outward and readily replaced endoscopically); and 1 abscess around the skin opening resolved by drainage (Table 3).

Two patients who had undergone SG in another center underwent combined endoscopic procedures (pigtails with glue apposition after T-tube placement). These patients had complex gastrocutaneous trans-diaphragmatic and gastrocutaneous bronchial fistula paths. Their primary endoscopic treatment with a T-tube had reduced the fistula diameter and created a gastrocutaneous path due to T-tube latex material activity. Their secondary treatment with pigtails followed by gluing facilitated effective treatment. The time between the bariatric procedure and T-tube placement for each patient, respectively, was 67 and 140 days; between the bariatric procedure and fistula closure, 360 and 323 days; and removal of the T-tube, 289 and 140 days.

One patient did not heal from her chronic LGCF with T-tube and endoscopic treatment. An abnormal hyperpressure in the bariatric construction (a medio-gastric twist on an SG) prevented fistula closure. After failure of several endoscopic treatments, the patient was treated by salvage laparoscopic fistulojejunostomy [14]. The fistula is currently closed.

## Discussion

Closure of large-diameter persistent postoperative leaks, particularly potentially life-threatening LGCFs, continues to be one of the most challenging complications of bariatric surgery with no agreed standardized approach. First-line response should be medical therapy and endoscopic techniques [2, 9, 15]. Double pigtail drains are the standard for internal drainage of small-orifice leaks [15]. Pigtails are at a high risk of migration (e.g., SG) or gastric erosion with long-term development of LGCFs (e.g., RYGB) where the leak orifice is ≥ 2 cm.

Given a high-pressure environment (e.g., SG sleeve), leaks are more problematic to seal even when effective drainage is achieved [14]. Large chronic leaks in this setting may persist, with extended closure times of 30 to 270 days [16, 17]. For these cases, our team developed a new strategy utilizing Kehr’s T-tube.

A well-described method of wound closure in management of esophageal perforation or blunt injury is T-tube placement in the esophagus through the injury site, creating a *controlled* esophagocutaneous fistula [18]. Leakage is thereby reduced locally and provides a drainage path through the skin to the outside of the body that averts sepsis and aids wound healing. In adapting the esophagocutaneous fistula model, we endoscopically placed the short arm of the T-tube in the gastric lumen to complete the positioning, directed the long arm of the T-tube through the conduit created by the spillage to the skin opening.

LGCF studies are extremely sparse, with < 50 reports within the last 10 years. One-third of these address LGCFs following bariatric surgery; fewer still describe post-bariatric procedure LGCFs treated with a T-tube. In 2010, Court et al. reported a post-SG staple-line disruption treated successfully with T-tube gastrostomy [19]. In 2013, El Hassan et al. placed a T-tube in 2 large post-SG staple-line leaks and 3 revisional AGB leaks [20]. In 2015, Barreca et al. reported 7 patients with post-SG staple-line leaks in which a T-tube was placed at the leak site by endoscopic-to-laparoscopic port insertion [21]. There appear to be no published sizeable bariatric surgery experiences of LGCFs treated with T-tubes to compare to the 14 LGCFs in the current study.

Our 92.9% T-tube closure rate in difficult LGCFs compares well with the clinical success rate of 78.5% using double pigtail drains in Donatelli et al.’s large experience of 285 post-SG fistulas *inclusive* of LGCFs treated endoscopically [19], and with Giuliani et al.’s global meta-analytic evaluation of 83.4% post-SG fistula closures using double pigtails [20]. In Okazaki et al.’.s 2018 meta-analytic summary of stent placement with 28 included studies, stents were effective in 73.0% of SG and 76.1% of RYGB fistula closures [22]. Generally, LGCF management with pigtails requires 2–3 endoscopic incursions to close; in the current study, T-tube placement achieved LGCF closure in 13/14 SGs and other bariatric procedures in 1–2 treatments. T-tube placement was an effective, low-morbidity, and well-tolerated treatment for persistent post-bariatric fistulas, especially LGCFs.

Each intervention generates more greater equipment utilization and hospital time [23]. The T-tube required a low added hospitalization time for fistula management (median 29.0 [8.0–49.0] days) with a cost of 8 Euros/each compared to a pigtail drain (80 Euros) or stent (800 Euros).

### Limitations

LGCFs can only be studied retrospectively as an unintended negative artefact of surgical procedures; a larger multicentric study sample would provide a more definitive test of this novel treatment.

### Conclusions

To our knowledge, the current study represents the largest sample to date of post-bariatric surgery LGCFs treated with Kehr’s T-tube. The main study finding is that, when standard endoscopic techniques failed, the novel and often life-saving T-tube technique was successful in resolving 92.9% (13/14) of large gastrocutaneous fistulas.
